# Small RNA-Seq Reveals the Effect of Formaldehyde Treatment on Chicken Embryo Liver microRNA Profiles

**DOI:** 10.3390/ijms262110633

**Published:** 2025-10-31

**Authors:** Saffet Teber, Mustafa Özdemir, Ghulam Asghar Sajid, Selma Büyükkılıç Beyzi, Mehmet Kizilaslan, Yunus Arzık, Servet Yalçın, Stephen N. White, Mehmet Ulas Cinar

**Affiliations:** 1 Department of Animal Science, Faculty of Agriculture, Erciyes University, 38280 Kayseri, Türkiye; saffetteber@erciyes.edu.tr (S.T.); mustafa.ozdemir@erciyes.edu.tr (M.Ö.); gasajid@cityu.edu.hk (G.A.S.); sbuyukkilic@erciyes.edu.tr (S.B.B.); 2 Department of Animal Science, Graduate School of Natural and Applied Sciences, Erciyes University, 38280 Kayseri, Türkiye; 3 Department of Infectious Diseases and Public Health, Jockey Club College of Veterinary Medicine and Life Sciences, City University of Hong Kong, Hong Kong SAR 999077, China; 4 Department of Animal and Dairy Sciences, University of Wisconsin–Madison, Madison, WI 53706, USA; kizilaslan@wisc.edu; 5 Department of Animal Science, Faculty of Veterinary Medicine, Aksaray University, 68100 Aksaray, Türkiye; yunus.arzik@aksaray.edu.tr; 6 Department of Animal Science, Faculty of Agriculture, Ege University, 35100 İzmir, Türkiye; servet.yalcin@ege.edu.tr; 7 Poultry Microbiological Safety & Processing Research, United States National Poultry Research Center, United States Department of Agriculture-Agricultural Research Service, Athens, GA 30605, USA; stephen.white@usda.gov; 8 Department of Veterinary Microbiology & Pathology, College of Veterinary Medicine, Washington State University, Pullman, WA 99164, USA; 9 Betül Ziya Eren Genome and Stem Cell Center, Erciyes University, 38280 Kayseri, Türkiye

**Keywords:** *Gallus gallus*, chicken embryo, miRNA, formaldehyde, expression profile

## Abstract

Formaldehyde (FA) is commonly used for hatchery disinfection, where it reduces microbial growth, ensures successful egg hatch and enhances healthy production, but its specific effects on embryonic development remain unclear. MicroRNAs (miRNAs) regulate gene expression post-transcriptionally and may mediate FA-induced transcriptional responses. Here, we investigated the impact of FA treatment on miRNA profiles in chicken embryo liver. Small RNA-seq libraries were constructed and sequenced using the Illumina NextSeq platform. Reads were trimmed and quantified using miRDeep2 version 2.0.0.3. Differential expression analysis was performed with DESeq2 (*p*-adjusted < 0.05 and |log_2_FC| > 1). Target genes of differentially expressed miRNAs (DEMs) were predicted with miRDB, and GO/KEGG/Reactome enrichment was conducted. Out of 662 total mature miRNAs detected, differential expression analysis identified 30 DEMs (11 up-regulated, 19 down-regulated). The highest fold increase was determined for gga-miR-3533 (log_2_FC = 4.45), and the most significant decrease was determined for gga-miR-133b (log_2_FC = −3.38). Pathway analysis revealed miRNAs affecting signaling pathways along with modules related to post-translational protein modification, immune system, and oxidative stress pathways. Our study demonstrates that FA treatment can affect critical biological processes by altering miRNA-mediated regulation in the developing embryonic liver and point to the need for functional validation of miRNA-target interactions to help determine mechanisms for FA benefits. Long term, these data may help serve as reference to identify new treatments with optimized response profiles.

## 1. Introduction

Formaldehyde (FA) is widely found in nature, industrial production, and consumer products [1]. In addition to being found indoors and outdoors, FA can be a natural product in most living systems [2,3]. FA has been widely used in the poultry industry to disinfect brooder houses, hatcheries [4], and feed [5]. FA may exert mutagenic and carcinogenic effects by inducing DNA damage and impairing DNA repair mechanisms [6]. Also, it can induce adduct formation and cross-linking by covalently binding to DNA and proteins [7]. One of the significant effects of FA exposure is the epigenetic modifications it induces in living organisms [8]. It is estimated that miRNAs, one of the epigenetic modifications, regulate up to 35% of formaldehyde-induced transcriptional responses [9]. Additionally, miRNAs that respond to FA exposure are likely regulators of critical pathways, including those related to immune/inflammatory response and apoptosis/proliferation [9]. Understanding the specific effects of FA on embryonic development, including its impact on critical regulatory molecules such as miRNAs, is vital for assessing its benefits and risks and optimizing its use [1].

Epigenetics describes inherited phenotypic changes that occur without a change in the DNA sequence [10,11]. Epigenetic mechanisms include DNA methylation, histone modification (which alters chromatin structure), and microRNAs (miRNAs) (which control gene expression and protein activity) [12]. miRNAs are small, non-protein-coding RNA molecules of approximately 21–25 nucleotides in length and are critical post-transcriptional regulators that bind to target messenger RNAs and suppress gene expression at the translational level or cause mRNA degradation [13]. miRNAs can control the expression of up to 30% of genes within a given genome [14]. A single miRNA can have one to several hundred target mRNAs [15]. These small molecules are considered critical post-transcriptional regulators of gene expression [13] because they mediate protein synthesis by binding to target mRNAs [16]. After being transported to the cytoplasm, single-stranded miRNAs bind to their complementary sequences in mRNA and inhibit translation, thereby promoting silencing of miRNA target genes [17]. This nonhomologous binding allows individual miRNAs to regulate hundreds of target genes [18].

The chicken embryo is also a robust animal model for studying epigenetic reprogramming processes. In vertebrates, critical epigenetic reprogramming events occur during early embryogenesis and germ cell development [19]. miRNA expression has been characterized in early developmental stages of chick embryos [15]. Because miRNAs are conserved across species, understanding when and where they are expressed in chick embryos will increase our current understanding of gene regulation during vertebrate development [15].

The liver plays a crucial role in the development of the chick embryo, particularly during key metabolic and physiological transitions. Throughout the embryonic period, the liver is primarily responsible for the metabolism of yolk lipids, which serve as the primary energy source for the embryo [20]. More than 90% of the energy in late-term embryonic development is derived from the oxidation of these yolk lipids, illustrating the liver’s central role in embryonic energy metabolism [21]. Furthermore, the liver synthesizes vital proteins and biochemical processes for the chick embryo’s development. For instance, during the last week of embryo maturation, significant absorption and oxidation activities related to yolk lipids occur [22]. This period is critical for progressing to metabolic independence post-hatch [23]. Notably, the liver’s enzymatic profile changes in response to the transition from embryonic to neonatal states, altering lipid metabolism and energy allocation essential for healthy development immediately after hatching [24]. In addition to its metabolic functions, the liver in chick embryos acts as a hematopoietic organ during specific development stages, contributing to blood protein synthesis, such as albumin, and playing a role in detoxifying potential teratogens or harmful substances [25].

In the poultry industry, losses due to microbial contamination of hatching eggs can reach millions of dollars [4]. Although FA is a widely used disinfectant in hatchery, the full range of its biological effects on developing poultry has not been evaluated. No molecular study in the literature shows the effect of FA treatment on miRNA of chicken embryos. This study aims to fill the research gap regarding the changes in miRNA profiles in chick embryo livers caused by using FA for pre-hatching disinfection.

## 2. Results

### 2.1. miRNAs Sequencing Data

A total of 6 small RNA libraries from the FA and control groups of chicken were used for miRNA sequence analysis. In the FA group, miRNA libraries yielded 21,156,913 raw reads. On the other hand, in the control group, miRNA libraries yielded 18,719,725 raw reads. After removing low-quality reads and adaptor sequences, over 33,381,401 clean reads were obtained from each of the 6 miRNA libraries (Table 1). The percentage of total mapped clean reads for each miRNA library was 72.3–93.6%, aligned with the chicken reference genome (bGalGal1.mat.broiler.GRCg7b). The GC content of 6 miRNA trimmed samples were between 42% and 46%, and Q30 percentages were >96.69% (Table 1).

Known conserved miRNAs were identified using miRBase (http://www.mirbase.org/ accessed on 14 January 2025). In this study, 662 mature miRNAs were analyzed. The 10 most abundant known miRNAs in each sample were gga-miR-122-5p, gga-miR-148a-3p, gga-miR-126-3p, gga-miR-143-3p, gga-miR-100-5p, gga-miR-21-5p, gga-miR-26a-2-5p, gga-miR-26a-5p, gga-miR-101-3p, and gga-miR-30e-5p.

**Figure 1:**
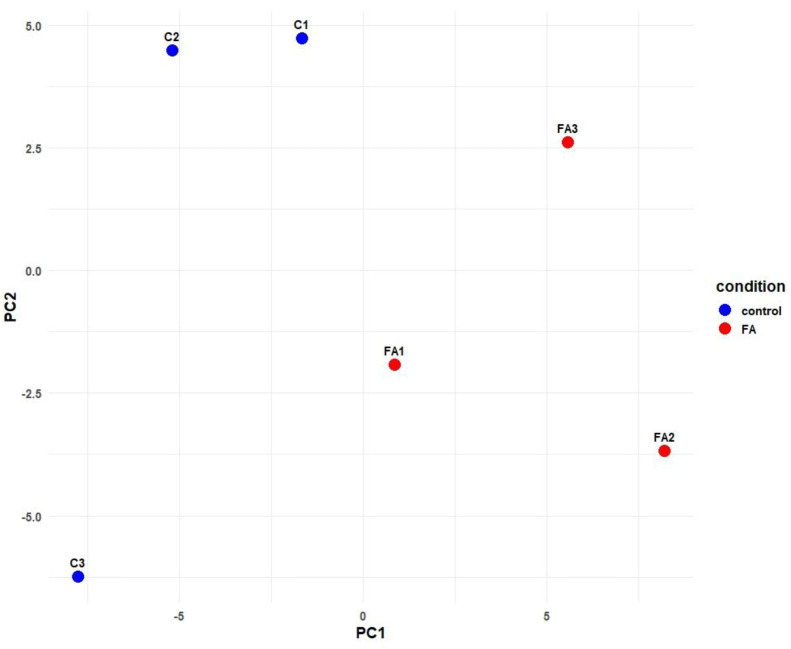
Principal Component Analysis (PCA) of miRNA expression profiles from chick embryo liver samples. Control samples (C1–C3, blue) cluster separately from formaldehyde-treated samples (FA1–FA3, red) along PC1, indicating a treatment effect.

Principal component analysis (PCA) of the miRNA expression profiles revealed a clear separation between FA-treated and control samples. In PCA, PC1 (41% variance) distinctly differentiated control and FA samples, validating the experiment’s repeatability and the significant impact of FA on global expression (Figure 1).

### 2.2. Differentially Expressed miRNA in the Chicken Between FA and Control

We performed differential expression analysis to identify miRNAs that were significantly altered by FA treatment with adjusted *p*-value < 0.05 and |log_2_FC| > 1. Of the 30 DEMs found, 11 miRNAs were up-regulated, and 19 miRNAs were down-regulated (Table 2). gga-miR-133b, gga-miR-133c-3p, gga-miR-205a, gga-miR-30a-3p, gga-miR-194, gga-miR-133a-5p, gga-let-7l-5p, gga-miR-133a-3p, gga-miR-1388a-5p show significant down-regulated differential expressions, while gga-miR-12214-5p, gga-miR-12239-5p, gga-miR-3533 show significant up-regulated differential expressions (|log_2_FC| > 2).

**Figure 2:**
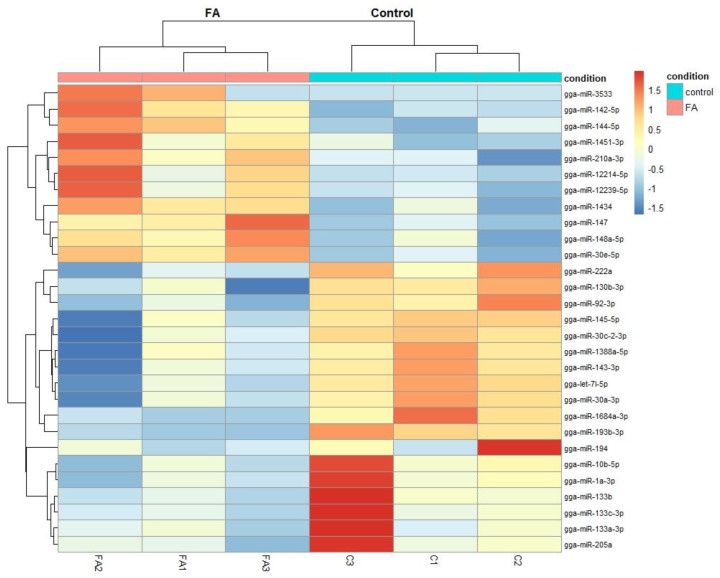
Heatmap of differentially expressed miRNAs. Each row represents significant DEMs, and each column is a sample (FA1, FA2, FA3 = FA-treated; C1, C2, C3 = control). The red color indicates higher level of expression, and the blue color indicates lower level of expression.

To visualize the differences in miRNA expression patterns, we generated a heatmap of the 30 DEMs (Figure 2). Significant differences in clustering were observed in miRNA expression profiles between the control and FA groups. Especially, gga-miR-3533 and gga-miR-133b showed the highest group differences. A volcano plot was used to visualize differential miRNA expression distribution. gga-miR-3533 (log_2_FC = 4.45) and gga-miR-133b (log_2_FC = −3.38) were notable for their statistical significance (*p*-adjusted < 0.05) and substantial fold change (Figure 2).

**Figure 3:**
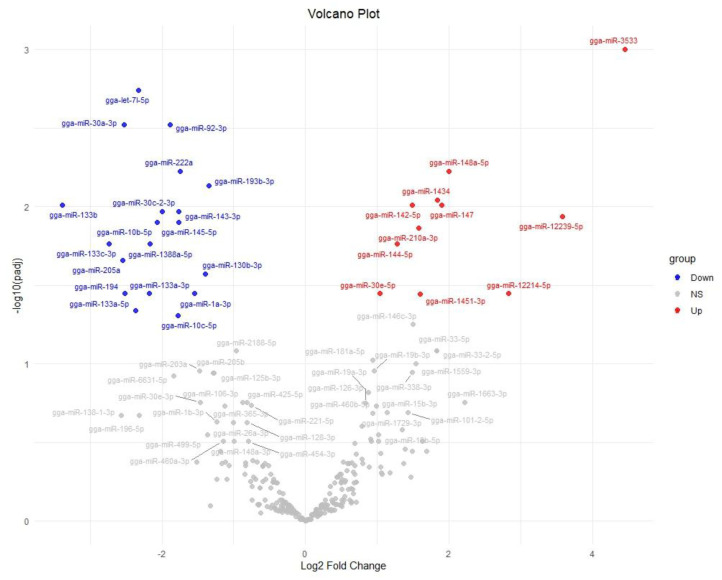
Volcano plot of differential miRNA expression results of FA-treated group vs. control group. Each point represents an miRNA; blue points indicate significantly down-regulated miRNAs in the FA-treated group, red points indicate significantly up-regulated miRNAs (*p*-adjusted < 0.05, |log_2_FC| > 1), and gray points are non-significant (NS).

We also used a volcano plot to summarize the statistical significance versus the change for all miRNAs (Figure 3). In the volcano plot, points corresponding to gga-miR-3533 and gga-miR-133b are far on the right (red) and left (blue), respectively, and are among the highest on the y-axis, confirming that these two miRNAs are both highly significant and have large fold changes. The plot also highlights other DEMs that did not significantly change (gray points).

### 2.3. Functional Enrichment and Pathway Analysis of Target Genes of Differentially Expressed miRNAs

Downstream of the miRNAs themselves, there were 2334 differentially expressed target genes for down-regulated miRNAs and 1032 target genes for up-regulated miRNAs (Table S1). These were employed to better understand the function of differentially expressed miRNAs through GO and KEGG pathway enrichment. The GO terms include biological processes (BP), cellular components (CC), and molecular functions (MF). Furthermore, the KEGG and Reactome pathways in differentially expressed miRNAs target genes with *p*-value < 0.05 were analyzed. For down-regulated miRNAs, 1839 were enriched based on gene ontology and 453 were enriched based on pathway, while for up-regulated miRNAs, 960 were enriched based on gene ontology and 170 were enriched based on pathway.

**Figure 4:**
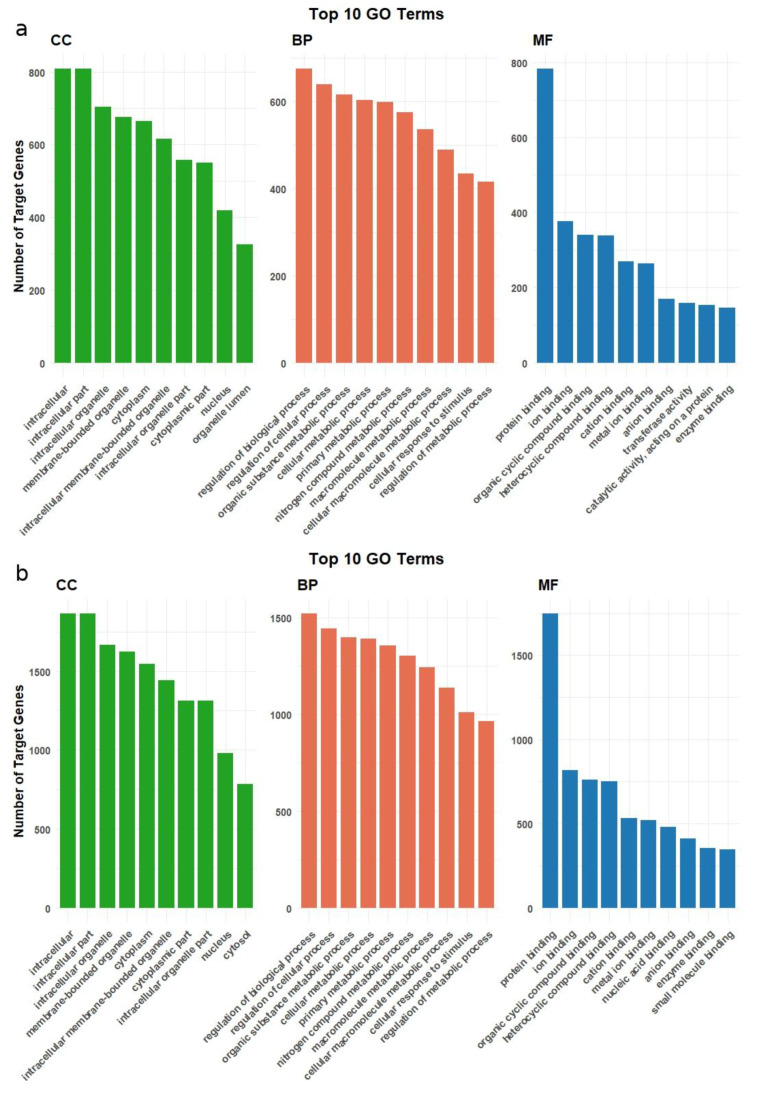
GO terms from Biological Process (BP), Cellular Component (CC), and Molecular Function (MF) categories are included in bar plot of DEMs target genes. (**a**) Top 10 Gene Ontology (GO) terms enriched among predicted target genes of FA-up-regulated miRNAs. (**b**) Top 10 Gene Ontology (GO) terms enriched among predicted target genes of FA-down-regulated miRNAs.

Gene Ontology enrichment revealed shared and distinct terms between the down- and up-regulated miRNA target genes (Table S2). Many fundamental cellular components and processes were enriched in both groups. However, some differences were found. Target genes of down-regulated miRNAs were markedly enriched. Within Cellular Component, they clustered in large intracellular domains—intracellular organelle membrane, cytoplasmic part, and related membrane-bounded organelle terms—while the Biological Process ontology was dominated by global control and metabolic categories such as regulation of biological process, cellular metabolic process, and macromolecule metabolic process. The target genes of downregulated miRNAs are largely similar to those target genes of up-regulated miRNAs, but there are differences. Among the 10 most enriched terms in CC, organelle lumen is not found among downregulated target genes but is among up-regulated target genes. Similarly, in the MF category, the terms transferase activity, catalytic activity, acting on a protein, and enzyme binding are only found among up-regulated target genes. Figure 4 shows the top 10 enriched GO terms for target genes of down- and up-regulated miRNAs across the CC, BP, and MF categories.

**Figure 5:**
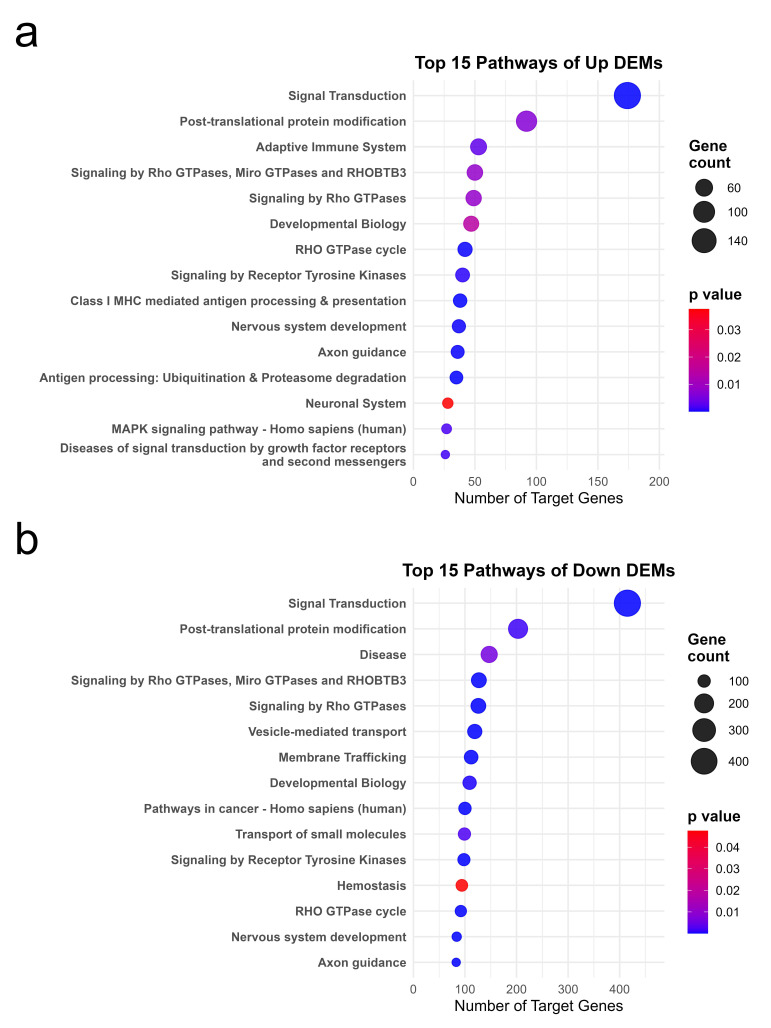
The pathway analysis for target genes of DEMs. (**a**) Top 15 enriched pathways (Reactome/KEGG) among predicted target genes of up-regulated miRNAs. Each pathway is plotted with the number of target genes (dot size) and enrichment *p*-value (color intensity). (**b**) Top 15 enriched pathways (Reactome/KEGG) among predicted target genes of down-regulated miRNAs. Each pathway is plotted with the number of target genes (dot size) and enrichment *p*-value (color intensity).

Pathway enrichment analysis showed further insights, especially regarding higher-level biological pathways related to FA treatment via miRNA regulation. Many enriched pathways were common to the down- and up-regulated target gene sets, including general signaling and metabolic pathways. Significant differences were observed in accordance with the above GO results. The target genes of down-regulated miRNAs displayed enrichment in several extensive pathway categories, including Signal Transduction (Reactome), Post-translational Protein Modification, Rho GTPase signaling pathways, and Disease pathways. On the other hand, the target genes of up-regulated miRNAs were found to be enriched in pathways including the adaptive immune system, antigen processing and presentation, and developmental biology, in addition to general signal transduction and protein modification pathways. Among the top 15 enriched pathways for up-regulated miRNA targets were multiple immune system pathways. Figure 5 shows the top enriched pathways (Reactome and KEGG) for targets of down- and up-regulated miRNAs.

## 3. Discussion

In this study, we used high-throughput miRNA sequencing technology to identify changes in the miRNA profile in chick embryo liver caused by using FA for pre-hatching disinfection. 662 miRNAs were detected by small RNA sequencing of six samples in chick embryo liver. The differential analysis identified 30 DEMs with a *p*-adjusted < 0.05, caused by the pre-hatching disinfection using FA. The findings indicate that FA can influence gene expression at the epigenetic level in embryonic tissues. Studies have demonstrated that exposure to formaldehyde can result in broad epigenetic modifications, including alterations in DNA methylation [26,27,28], histone modifications [29,30], and changes in miRNA expression [9,31,32,33]. Park et al. [34] reported that, in Yucatan minipigs, FA exposure suppresses the immune response, as evidenced by changes in the distribution of helper T cells and expression levels of immune-related factors.

It has been observed that miRNAs with different expression in embryos exposed to FA target signaling pathways that are critical for normal embryonic development. Liao et al. [35] identified 2459 miRNAs in the early stages of chicken embryos, revealing stage-specific miRNA profiles and demonstrating that many of these are key nodes in essential pathways such as VEGF, Insulin, ErbB, MAPK, Hedgehog, TLR, and Hippo. Those pathways that regulate organogenesis and differentiation processes are tightly regulated for the healthy development of the embryo. In our study, among the predicted target genes of the miRNAs altered due to FA treatment, many pathways were found related to cell proliferation, differentiation, and apoptosis. For example, our enrichment analyses have shown that pathways associated with developmental processes, such as Wnt and TGF-β signaling pathways, are affected in the FA group. Xu et al. [36] examined the miRNA profiles in chicken granulosa cells throughout follicle development and reported that 48 miRNAs, which showed dynamic changes over time, regulated hundreds of target genes involved in processes such as oocyte meiosis, progesterone-induced oocyte maturation, Wnt, and TGF-β signaling. In the same study, it was shown that some miRNAs associated with apoptosis and autophagy (for example, the let-7 family, gga-miR-363-3p, gga-miR-30c-5p, and gga-miR-21-5p) play critical roles during follicle development. Also, gg-miR-30a-3p was reported to be associated with myoblast proliferation and differentiation [37]. Our findings also suggest that some miRNAs, such as gga-miR-30a-3p, which decrease with FA exposure, normally control the balance between cell death and survival. In this context, it is conceivable that FA could disrupt embryonic organ development by creating an imbalance in such developmental pathways.

Among the differentially expressed miRNAs caused by FA, there are some important regulators that are known to be tissue or process-specific under normal conditions. For example, in our study, a significant decrease in the levels of gga-miR-133b and gga-miR-133c-3p was observed with FA exposure. The miR-133 family plays a key role in the development and differentiation of skeletal muscle [38]. Shi et al. [39] demonstrated that thousands of miRNAs are expressed during embryonic muscle development, and specifically, pathways associated with muscle development, such as Wnt, Notch, and MAPK, are targeted by these miRNAs. It remains to be determined whether FA alteration of miR-133s improves or impairs muscle development in detectable ways. MiR-10 b-5p is involved in several disease processes, such as cancer and neurological disorders [39,40,41]. Recent studies indicate that miR-10b-5p expression varies in spinal cord injury (SCI) models; its down-regulation is associated with heightened neuronal apoptosis and impaired tissue repair [42]. Overexpression of miR-10b-5p has been demonstrated to promote autophagy and inhibit apoptosis via UBR7 inhibition of the Wnt/β-catenin signaling pathway [43]. In our study, the observed down-regulation of miR-10b-5p in FA treated embryonic liver suggests potential alterations of as yet undefined magnitude in cellular stress, apoptosis, or developmental processes. Similarly, another miRNA that significantly decreased in the group treated to FA, miR-205a, was demonstrated in a study on Tibial dyschondroplasia (TD, a kind of bone metabolic disease in fast-growing broilers, which seriously restricts the development of the poultry industry) in chickens where the transfection of an miR-205a overexpression plasmid reduced chondrocyte growth and development in TD while enhancing apoptosis; conversely, blocking miR-205a had opposite effects [40]. In another study in chickens, it was reported that miR-205a can directly bind to the 3′UTR of *CDH11* and that the overexpression of miR-205a could inhibit proliferation in both QM7 and CPM cell lines, while at the same time promoting myoblast differentiation [41]. Moreover, given the demonstrated role of miR-205a in targeting *CDH11*, its reduced expression may alleviate the suppression of *CDH11*, potentially altering cell–cell adhesion and tissue remodeling dynamics [41]. Also, Ouyang et al. [42] reported that miR-205a are key growth-related target genes in the network. In our study, the suppression of gga-miR-205a by FA suggests that FA treatment could also affect skeletal system development. While numerous studies have shown that FA fumigation has beneficial effects on disinfection and no major detrimental effects on growth [4,5], our study informs an overall perspective that there may be room for further improvement in treatment options. Furthermore, our data provide a reference against which to compare future treatment options.

Our analyses showed that a significant portion of the target genes of the miRNAs altered by FA exposure are related to immune response and inflammation processes. This situation suggests that FA may affect immune system development and immunological balance during the embryonic development period. Indeed, it is well known that environmental stress can strongly alter miRNA profiles in infection models. We also observed the down-regulation of gga-miR-194 in FA-treated embryos. Jia et al. [43] reported that deep sequencing analyses under APEC infection reported that miR-194 showed significant changes among different experimental groups. This study shows that gga-miR-194 is among the critical miRNAs that may play a role in host immune response and modulate processes such as inflammatory signaling and apoptotic regulation. Also, Dinh et al. [44] highlighted the differential expression of gga-miR-194 in chicken lines showing different disease susceptibility. In chicken jejunum infected with *Eimeria maxima*, gga-miR-1388a-5p was also among the set of miRNAs consistently down-regulated at both 4 d and 7 d post-infection [45]. Moreover, network analysis of this miRNA showed that it may be involved in the immune response to *Eimeira maxima* infection in the jejunum. These findings indicate that biological stressors, such as infections, lead to widespread miRNA-mediated reprogramming in the host. In our study, the number of miRNA changes detected in embryos exposed to FA remained at a more modest level compared to infection models (a total of 30 miRNA changes), which may suggest that the chemical stressor FA elicits a more limited but specific molecular response.

In contrast to the suppression of many miRNAs, some miRNAs were up-regulated in response to FA. Three miRNAs in particular—gga-miR-12214-5p, gga-miR-12239-5p, and gga-miR-3533—were significantly up-regulated with log_2_FC > 2. These miRNAs might be part of an adaptive regulatory response to FA stress. A comprehensive study on chicken muscle development revealed that more than 200 mRNA molecules were indirectly controlled by a competitive endogenous RNA (ceRNA) network comprising seven miRNAs, including gga-miR-12239-5p [46]. This network’s target genes include stress response proteins and glycolysis enzymes, indicating that gga-miR-12239 could be involved in energy metabolism and muscle development processes [46]. In a study reported on miRNA expression patterns following astrovirus infection in chickens, gga-miR-3533 was one of the miRNAs most highly expressed in the spleens of infected birds [47]. On the other hand, a study on ethanol exposure in chicken embryos found that gga-miR-3533 was notably downregulated by alcohol exposure [48]. These results underline how important molecular mediators in the response of the embryonic liver to FA treated stress could be miRNAs. Their significant up-regulation indicates the activation of conserved pathways linked to metabolic adaptation, cytoskeletal organization, and immune modulation. Functional enrichment analysis of the expected targets of up-regulated miRNAs also showed overrepresentation of GO terms such as nuclear lumen, cell development, immune system process, and transferase complex, indicating a change toward immune and stress-adaptive responses. Pathway analysis also revealed more signaling modules, including the MAPK pathway, antigen processing and presentation, and adaptive immune system, all pointing to increased cellular activity connected to damage recognition, repair, and inflammation. Emerging evidence suggests that embryonic miRNA dysregulation can have lasting effects on organ development, immune function, and metabolic programming in poultry and livestock [49].

Our study demonstrates that FA can potentially lead to important effects by reshaping miRNA-mediated gene regulation during the embryonic period. The miRNA changes detected in the embryonic liver affect a wide range of target gene pools, from epigenetic regulatory genes to developmental signals, metabolic pathways, and immune responses. This situation indicates that the phenotypic outcomes that may arise in the embryo as a result of FA exposure can be multifaceted and highlight the need for further research to understand the long-term effects of hatchery disinfection practices.

## 4. Materials and Methods

### 4.1. Animal Ethics

This study was approved by the Institutional Animal Care and Use Committee of Erciyes University, Kayseri, Türkiye (4 January 2023, #004). This research protocol was in accordance with the guidelines of the Turkish Council on Animal Experimentation for agricultural animal facilities (15 February 2014, #28914).

### 4.2. Experimental Design and Sample Collection

The hatching eggs in this research were Tinted breed chick embryos procured from Berrin Enez Hathery and Layer Co. (Balıkesir, Türkiye) and placed in two separate incubators (Petersime NV, Zulte, Belgium). The eggs were randomly allocated to two groups. FA was not administered to half of the embryos, which were retained as controls. The remaining half of the eggs (*n* = 75 eggs) were subjected to FA by combining formalin and potassium permanganate (KMnO_4_) to release FA gas. The heat necessary for the formalin release was produced by combining 35 mL of formalin with 17.5 g of KMnO_4_ [50]. The process involved the combustion of FA, egg disinfection, and gas evacuation, which required 25 min [51]. The FA concentration was 6 g/m^3^. The incubator was not subjected to any additional disinfection procedures during the investigation. During the fumigation process, the relative humidity was maintained at 75% and the temperature was maintained at 24–25 °C. A total of 150 fertilized chicken eggs were incubated at a temperature of 37.8 °C and a relative humidity of 58–63%. The embryos were acquired by fracturing the eggs on the 18th day of incubation and were euthanized using the cervical dislocation method. This time point was chosen as it corresponds to a developmental stage where organogenesis and morphology are largely complete, as defined by Hamburger and Hamilton [52]. Subsequently, liver samples were obtained and snap-frozen in liquid nitrogen and subsequently stored at −80 °C until small RNA extraction was conducted. Six developed chick embryos were selected randomly (n = 3 from each group).

### 4.3. RNA Extraction and Quality Control

Total RNA was extracted from six liver tissues utilizing an RNA extraction kit (Nucleogene, Istanbul, Türkiye) following the manufacturer’s instructions. The purity and concentration of RNA were evaluated using the BioSpec-Nano Spectrophotometer (Shimadzu, Kyoto, Japan). The quality of total RNA was assessed using the RNA Integrity Number (RIN) using the Agilent Bioanalyzer 2100 system (Agilent Tech., Santa Clara, CA, USA) prior to cDNA library construction. The RINs of the samples ranged from 7.8 to 10.0.

### 4.4. Small RNA Library Preparation and Sequencing

Six libraries were established, comprising three from the FA and three from the control groups. Small RNA libraries were constructed using the NEBNext^®^ Multiplex Small RNA Library Prep Set for Illumina^®^ (New England Biolabs, Ipswich, MA, USA) following the manufacturer’s instructions and subsequently sequenced at the Novogene Bioinformatics Institute (Beijing, China) on an Illumina NextSeq 500 platform (Illumina, San Diego, CA, USA) following the vendor’s protocol with 50 bp single-end mode.

### 4.5. MiRNAs Sequence Data Analysis

Raw data assessed by FastQC (https://www.bioinformatics.babraham.ac.uk/projects/fastqc/ accessed on 9 December 2024). Then, cutadapt [53] was used for quality control trimming, and the adapter sequence was removed. Trimmed clean reads between 18 and 35 nt were maintained for downstream analysis. GC content and Q20/30 values were calculated using fastp [54]. Clean reads were mapped to the indexed by bowtie2 [55] reference chicken genome (Gallus_gallus.bGalGal1.mat.broiler.GRCg7b) using the mapper algorithm in the miRDeep2 package [56] with the allow-one-base mismatch parameter. For the analysis and quantification of known miRNAs, a species-specific reference set was prepared using the miRDeep2 software package. First, all mature miRNA sequences were downloaded from the miRBase database (version 22.1; http://www.mirbase.org/ accessed on 9 December 2024). Subsequently, the “extract_miRNAs.pl” module from the miRDeep2 core package was utilized to extraction only the sequences belonging to *Gallus gallus* (gga). This curated set of chicken-specific miRNA sequences then served as the exclusive reference for all downstream quantification analyses.

The raw data of this research were submitted to the NCBI Gene Expression Omnibus (GEO) (http://www.ncbi.nlm.nih.gov/geo/, accessed on 9 December 2024) under accession number GSE295531.

### 4.6. Differentially Expressed miRNAs Analysis

The expression levels of known mature miRNAs in each sample were counted from the created matrix from the 3 FA-treated and 3 control samples. The DESeq2 R package (version 1.49.9) was used to examine the differences in the expression levels between DEMs treated with formaldehyde and control groups with raw counts. The threshold of significantly different expression of miRNAs was set as *p*-adjusted < 0.05 (using BH method [57]) and |log_2_(fold change)| > 1.

### 4.7. Target Gene Prediction and Functional Analysis of DEMs

An in silico method, miRDB (http://www.mirdb.org/, accessed on 9 December 2024), was employed to predict the target genes of DEMs, with chicken database selected as the reference species. To mitigate false-positive target predictions, genes with a target score more than 80 were selected. Gene ontology (GO) enrichment and pathway (KEGG and Reactome) analysis were applied separately down/up miRNAs to the target gene candidates of DEMs using the ConsensusPathDB web service (Max Planck Institute, http://cpdb.molgen.mpg.de/, accessed on 9 December 2024) [58]. Biological process (BP), cellular component (CC), and molecular function (MF) terms in the GO database were obtained. KEGG and Reactome pathway services were used for pathway enrichment analysis. The R package ggplot2 was used for the GO and pathway visualization.

## 5. Conclusions

This study demonstrates that formaldehyde (FA) treatment during embryonic development significantly alters miRNA expression profiles in chicken liver. The regulation of both up- and down-regulated miRNAs suggests that FA disrupts target genes that support normal development.

Further studies should be conducted in the future to confirm the functional significance of the identified miRNA-target gene interactions. Additionally, additional studies are required to assess the persistence and phenotypic consequences of these epigenetic changes beyond the embryonic stage. Our findings highlight the epigenetic sensitivity of the developing embryo to commonly used environmental toxicants such as FA. These data also highlight the importance of reevaluating routine chemical applications in poultry production systems and the need for broader regulatory limits on FA treatment.

## Figures and Tables

**Table 1:** Quality and mapping summary of small RNA sequencing reads from control and FA-treated embryo liver samples.

	Raw Reads		Trimmed Reads					
Sample	Raw Reads	% GC	Trimmed Reads	% GC	Q20	Q30	Mapped Reads	Mapped %
C1	6,256,428	50%	5,377,299	46%	99.24%	97.32%	3,865,384	72.379
C2	6,974,290	49%	6,257,434	44%	99.24%	97.43%	5,181,287	83.227
C3	5,489,007	49%	4,993,507	44%	99.27%	97.49%	4,357,515	87.961
FA1	10,220,093	48%	8,814,039	43%	99.33%	97.71%	7,511,638	87.132
FA2	5,919,854	48%	3,820,899	42%	99.12%	96.97%	3,399,960	93.602
FA3	5,016,966	50%	4,118,223	46%	99.05%	96.69%	3,309,574	81.247

**Table 2:** Significantly differentially expressed miRNAs in FA-treated vs. control embryo livers.

miRNA	baseMean	Log_2_FC	*p*-Value	*p*-Adj
gga-miR-3533	38.164892	4.453057	0.000003	0.000997
gga-miR-12239-5p	13.882909	3.582699	0.000577	0.011505
gga-miR-12214-5p	10.217137	2.826239	0.003372	0.035794
gga-miR-148a-5p	1205.717617	1.994005	0.000121	0.005943
gga-miR-147	66.211133	1.893574	0.000335	0.009736
gga-miR-1434	77.881529	1.838995	0.000260	0.009074
gga-miR-1451-3p	73.627625	1.600179	0.003596	0.035836
gga-miR-142-5p	396.305198	1.485202	0.000382	0.009736
gga-miR-30e-5p	64,373.223801	1.035841	0.003019	0.035794
gga-miR-210a-3p	284.758497	1.580432	0.000832	0.013660
gga-miR-144-5p	725.246217	1.271770	0.001146	0.017249
gga-miR-30c-2-3p	190.978155	−1.994259	0.000499	0.010709
gga-miR-10b-5p	367.784900	−2.067845	0.000711	0.012542
gga-miR-193b-3p	447.476617	−1.344009	0.000183	0.007331
gga-miR-130b-3p	9184.792929	−1.393183	0.002106	0.026710
gga-miR-1a-3p	11,719.852639	−1.542965	0.003463	0.035794
gga-miR-222a	2048.361237	−1.740781	0.000127	0.005943
gga-miR-143-3p	121,035.324778	−1.762389	0.000463	0.010709
gga-miR-145-5p	8553.054840	−1.764542	0.000719	0.012542
gga-miR-10c-5p	55.671853	−1.772399	0.005286	0.049166
gga-miR-92-3p	3139.439756	−1.886186	0.000043	0.003015
gga-miR-1388a-5p	350.525535	−2.168952	0.001207	0.017249
gga-miR-133a-3p	236.212541	−2.173803	0.003326	0.035794
gga-let-7l-5p	396.995234	−2.322389	0.000013	0.001814
gga-miR-133a-5p	47.417866	−2.362333	0.004741	0.045612
gga-miR-194	43.556299	−2.514918	0.003255	0.035794
gga-miR-30a-3p	482.244321	−2.530401	0.000036	0.003015
gga-miR-205a	59.202319	−2.547512	0.001648	0.021905
gga-miR-133c-3p	42.136850	−2.731904	0.001236	0.017249
gga-miR-133b	28.789600	−3.383927	0.000383	0.009736

## Data Availability

Sequence datasets were submitted to the NCBI (National Center for Biotechnology Information) Gene Expression Omnibus (GEO) and are available under the accession number GSE295531.

## Supplementary Materials

The following supporting information can be downloaded at https://www.mdpi.com/article/10.3390/ijms262110633/s1.

## Abbreviations

The following abbreviations are used in this manuscript:

FA	Formaldehyde
DEMs	Differentially expressed miRNAs
PCA	Principal component analysis
GO	Gene Ontology
KEGG	Kyoto Encyclopedia of Genes and Genomes
